# Why does understanding the biology of fibroblasts in immunity really matter?

**DOI:** 10.1371/journal.pbio.3001954

**Published:** 2023-02-06

**Authors:** Zhi Yi Wong, Eloise Nee, Mark Coles, Christopher D. Buckley

**Affiliations:** Kennedy Institute of Rheumatology, University of Oxford, Oxford, United Kingdom

## Abstract

Fibroblasts are known for their ability to make and modify the extracellular matrix. However, there is more to them than meets the eye. It is now clear that they help define tissue microenvironments and support immune responses in organs. As technology advances, we have started to uncover the secrets of fibroblasts. In this Essay, we present fibroblasts as not only the builders and renovators of tissue environments but also the rheostat cells for immune circuits. Although they perform location-specific functions, they do not have badges of fixed identity. Instead, they display a spectrum of functional states and can swing between these states depending on the needs of the organ. As fibroblasts participate in a range of activities both in health and disease, finding the key factors that alter their development and functional states will be an important goal to restore homeostasis in maladapted tissues.

## Abstract 2

Fibroblasts have traditionally been thought of as structural cells, but technological advances have started to uncover important roles for fibroblasts in immune function. This Essay looks at why understanding the biology of fibroblasts really matters for health and disease.

## Introduction

**Fig 1:**
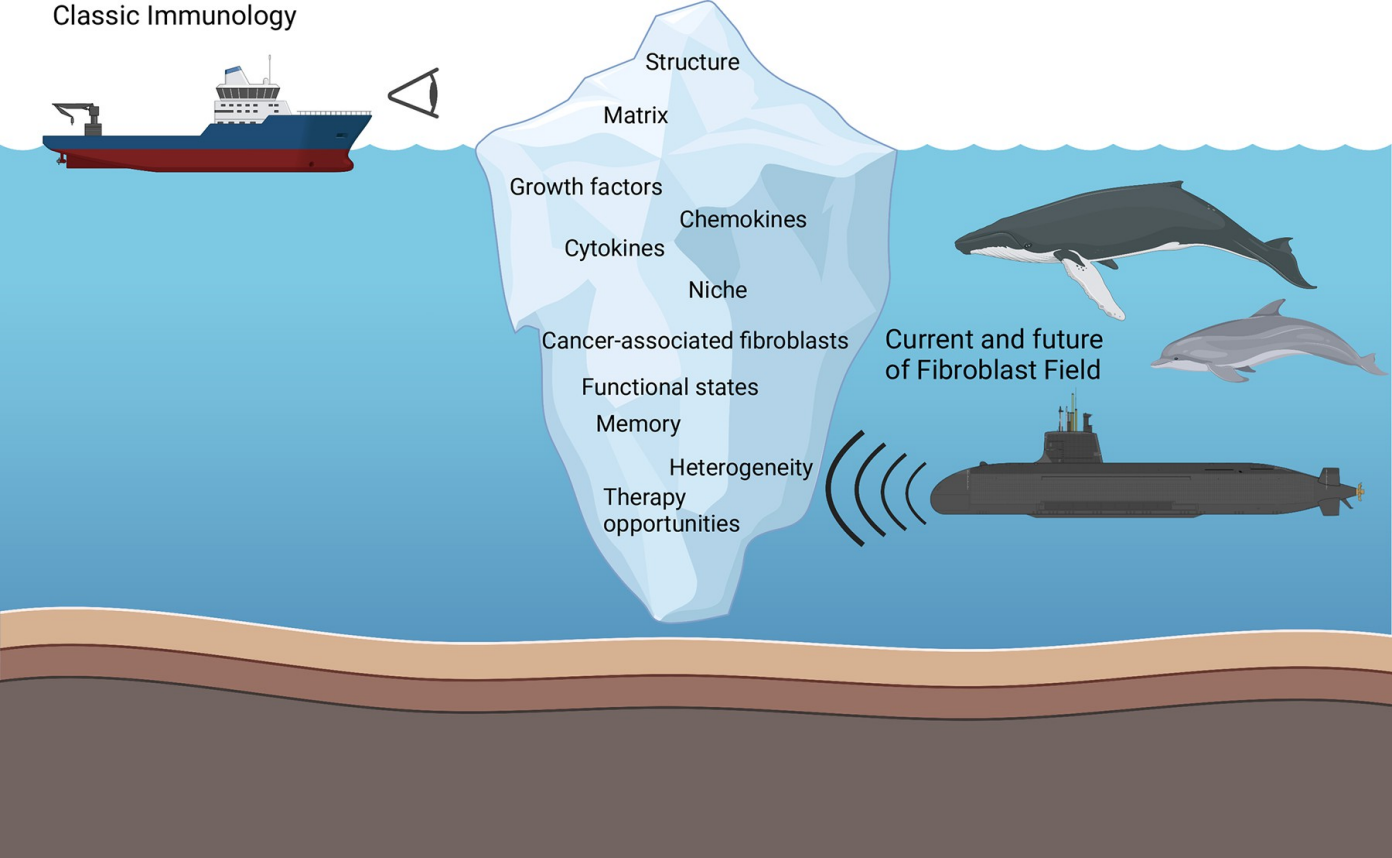
The past, present, and future of fibroblasts. Classical immunology generally views fibroblasts as the cells that provide structure and stiffness to tissues. In fact, that is only the tip of an iceberg and now that technologies have advanced (represented by the submarine with sonar), we have finally learnt that fibroblasts are cells of many talents that interact with cells within the tissue microenvironment (represented by the whale and dolphin). Figure is created with BioRender.com.

In the 19th century, Rudolf Virchow and Mathias Duval first described fibroblasts as the most common cell type of connective tissues. Fibroblasts were widely regarded as featureless cells with a spindle-shaped morphology. This view has persisted for many years [1,2]. Classic immunology textbooks do not normally have much to say about fibroblasts. Even if they do, fibroblast function is limited to making and modifying extracellular matrix (ECM) (Glossary) and their contribution to healing and repair (Fig 1). Major discoveries such as antibodies and the major histocompatibility complex (MHC), and the ease of access to blood as a source from which to study immune function, have led to huge volumes of research on cells of the hematopoietic system, thereby distracting the immunology field from the role that fibroblasts have in regulating immune cells in tissues.

## Glossary

### Extracellular matrix

A network on noncellular components, including fibrous proteins and growth factors, which supports cells and gives structure to tissues.

### Single-cell RNA sequencing

The quantitative analysis of the RNA transcriptome of individual cells, often from heterogenous tissues.

### Chemokines

A family of small cytokines and signaling proteins that control the migration and spatial localization of leucocytes, including CXCL13, CCL19, and CCL21.

### Cytokines

A broad category of small proteins that are involved in both anti-inflammatory and pro-inflammatory signaling, including TNF and IL-6.

### Lymphoid tissues

Specialized immune tissue including bone marrow, spleens, lymph nodes, and mucosal-associated immune tissues.

### Peripheral tissues

Peripheral tissues encompass tissues that are not specialized immune tissue.

### Tertiary lymphoid structures

Lymphoid-like structures that can organize and develop de novo in the periphery and they are associated with many diseases of chronic inflammation. For more information, see [3–5].

### Rheumatoid arthritis

A chronic inflammatory condition primarily affecting the synovium of specific joints in the body. For more information, see [6,7].

### Inflammatory bowel diseases

A heterogenous grouping of chronic inflammatory diseases affecting the gut, including Crohn’s disease and ulcerative colitis. For more information, please see [8–10].

### Cancer-associated fibroblasts

Fibroblasts that form part of the tumor microenvironment. For more information, see [11,12].

### Myofibroblasts

An activated fibroblast state characterized by expression of α-smooth muscle actin and contractile capability, associated with fibrosis across multiple organs and wound healing.

Let us step back a little bit and consider what a fibroblast really is. As the most abundant cell type in the connective tissue, it is derived from the embryonic mesenchyme. However, the field of cell biology has struggled to adequately define the fibroblast cell linage. Cells are generally considered to be fibroblasts by a rather negative description, when they are negative for epithelial, endothelial, and leucocyte markers and possess an elongated morphology. Unlike CD45 as a marker for leucocytes, we lack a universal marker specifically expressed by fibroblasts. Instead, a group of markers is normally used, which include podoplanin (PDPN), platelet-derived growth factor receptor alpha (PDGFRα), vimentin, α-smooth muscle actin, CD34, and CD90. The fact that there is no specific fibroblast marker might mean that the field has yet to identify it because we have not got the appropriate tools, or it might reflect the fact that fibroblasts are just not as universally distributed across the body in the way that leucocytes are. A critical question remains: Will we be able to map all the fibroblast lineages in the same way that hematologists have done for the cells of the hematopoietic system?

Previously, genome-wide profiling of genes using microarray and publicly available databases from The Immunological Genome Project (ImmGen) have revealed valuable insights, including transcriptional profiles of lymph node stromal cells in inflamed and resting states [13], regulatory networks of immune cells [14], and site-specific variations in fibroblast gene expression related to their positional identities [15]. Now, fate mapping of the fibroblast lineage has begun, driven by new technologies. Single-cell RNA sequencing (scRNA-seq) (Glossary) is one of the key enabling technologies that drives interest in and our understanding of fibroblasts (Table 1). Transcriptomic analysis enables the identification of fibroblasts without requiring any predefined markers. Fibroblasts can now be identified by modules of gene expression and compared across tissues, bypassing the need for a prerequisite universal marker. Within the fibroblast lineage, a contender for a “progenitor-like” population has started to emerge in the form of the Pi16-expressing fibroblast [16]. Technologies such as advanced imaging methods and open source bioinformatic tools leveraging advances in machine learning from across disciplines are now driving an increased understanding of the importance of fibroblasts in biology.

**Table 1:** Key technologies that enable the study of fibroblasts.

Method	Description	Uses
Minimally invasive tissue biopsy methods	Sample collection from patients with diseases before death. Enables far more reliable samples to be collected, including from patients during specific stages in the pathway of a disease.	Sampling synovium from patients with arthritis, colon from patients with inflammatory bowel disease, and skin from patients with vitiligo.
Single-cell multiomics	Methods are varied but include analysis of the transcriptome, proteome, and epigenome of individual cells. Examples of methods include scRNA-seq, CITE-seq, CyTOF, and ATAC-seq.	Uncovering the heterogeneity of fibroblasts within and between tissues and changes in fibroblast functional states.
Imaging techniques	Multiplex imaging and RNA probe-based imaging.	Uncovering the heterogeneity of fibroblasts within and between tissues and spatial organization of fibroblasts and their respective niches.
Mouse models	Transgenic mice targeting fibroblast subsets, Cre recombinase transgenic mice crossed to fluorescent reporter strains, delete strains, or floxed genes	Functional experiments show mechanism and causality, which need mice. Fate mapping, lineage tracing, and developmental studies.
Machine learning and high-dimensional analysis methods	Novel methods for analysis of high-dimensional data have accelerated in the field. Driven by requirements from single-cell data and imaging data. More modern methods are starting to come to the fore, for instance, tensor-based methods.	Integration of datasets across tissues, species, and modalities. Identifying interacting cells from sequencing data.
Open-source data and tools	Major platforms include Github, the human cell atlas, EBI, and many others.	A major enabling technology for analysis of complex data (for instance, multiomics). Also enables the creation of “atlases” from multiple datasets through data distribution.

The field of immunology research has evolved from a rather leucocyte-centric view to embrace a more extended immune system in which an understanding of tissue microenvironments and mucosal barrier function are now recognized as fundamental elements that determine immune responses. As technologies have advanced, we have learnt that fibroblasts are in fact a part of our immune system [17]. They are cells that convey site specificity and act as modulators of the induction, perpetuation, and resolution of an immune response (Fig 1). Fibroblasts provide supportive functions so that immune cells can drive appropriate responses to danger or damage signals. However, we are starting to learn that fibroblasts exist in distinct functional states and define the size and the function of the niches they occupy [13,16,18,19]. Do fibroblasts just play a supportive role, or are they the main cell type that set the scene for immune cells?

In this Essay, we review the recent progress in technologies that has advanced our understanding on the role of fibroblasts in our immune system. We discuss location-specific functions that fibroblasts perform and the relationship between fibroblasts and neighboring cells in different niches across physiological states. Fibroblasts can maintain homeostasis but also contribute to diseases when they go rogue, and as they are plastic in functional states, can we restore the balance as we treat diseases?

### Fibroblasts—Cells of many talents

All fibroblasts generate ECM and provide architectural support: a feature that is common to their lineage. However, not all fibroblasts are the same. Single-cell transcriptomic data show that there are common fibroblast types found across multiple organs but there are also those that are organ specific. Fibroblasts that are shared across tissues may represent a progenitor-like and a universal structural population [16]. Clear distinctions between chemokine-expressing (Glossary) lymphoid fibroblasts and specialized peripheral fibroblasts have also been described [16].

**Fig 2:**
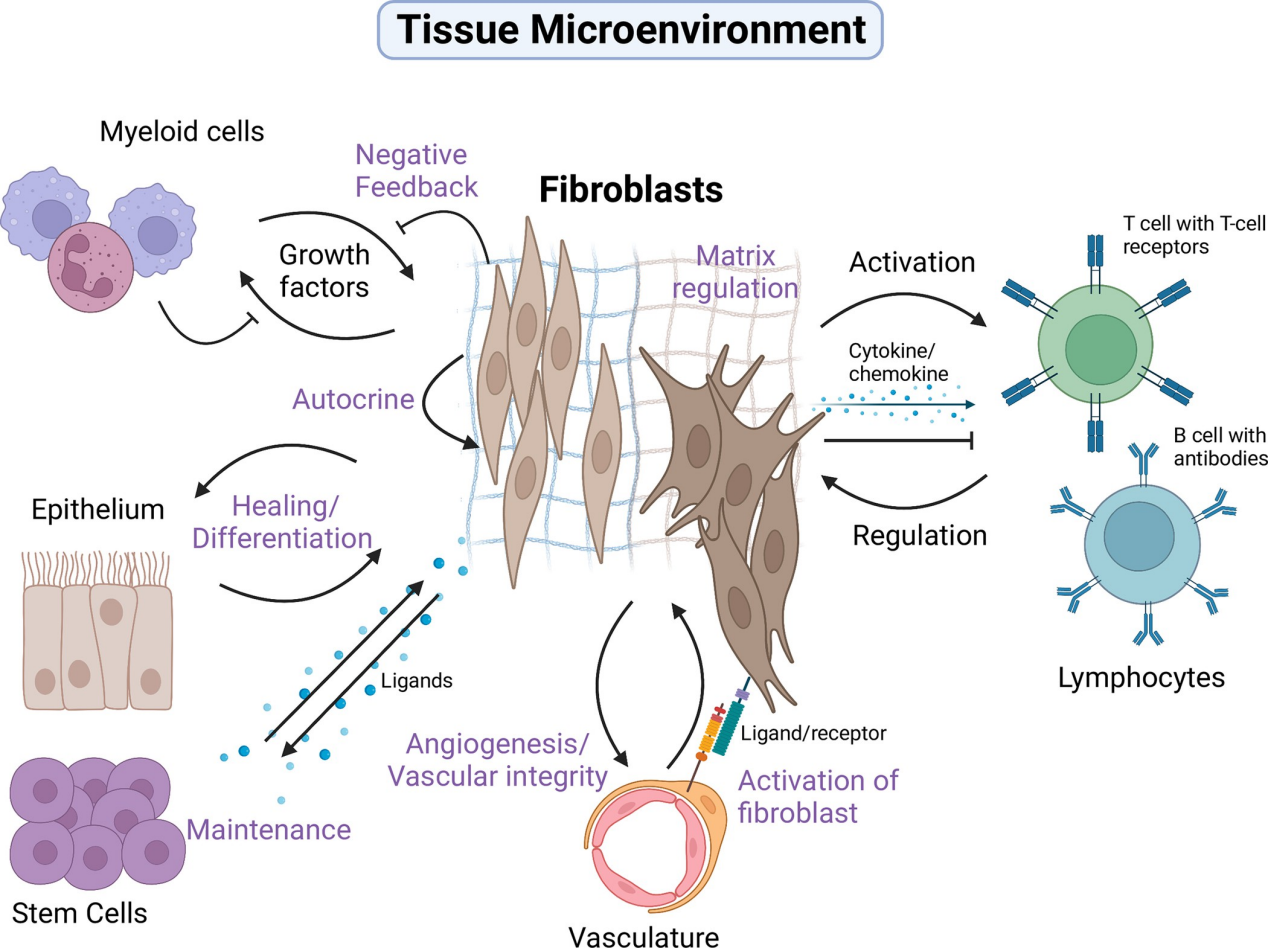
Fibroblasts are cells of many talents. All fibroblasts produce extracellular matrix and provide structural support. They form specialized partnerships with different cells depending on their tissue microenvironment. It is important to remember that fibroblast functional states are plastic and can be skewed or activated but this is highly contextual. Figure is created with BioRender.com.

Fibroblast functions exist as a spectrum rather than distinct subtypes, and their functions can be tipped towards different ends of a spectrum depending on the tissue context (Fig 2). Fibroblasts are specialized at interacting with different cells in tissue microenvironments. They build, maintain, and repair the infrastructure within the tissue microenvironment. Fibroblasts constantly sense the environment and crosstalk with macrophages, forming a stable circuit that supports each other by providing growth factors [20]. On the other hand, fibroblasts in an epithelial niche promote healing, support epithelial differentiation, and help maintain the epithelial stem cell niche [21]. Interaction between vasculature and perivascular fibroblasts enhances blood vessel integrity and stabilization [22].

Lymphocyte-interacting fibroblasts display high levels of crosstalk with T cells and B cells. They can recruit lymphocytes, constrain their functions, present antigens, and, when activated, express inflammatory chemokines and cytokines (Glossary). The activation and regulation of the immune system by fibroblasts is highly contextual [23]. Generally, fibroblasts in a homeostatic state are “peacekeepers.” They provide a tolerogenic threshold to dampen unnecessary immune responses. If activated, they can form amplificatory feedback loops and contribute to disease persistence [24]. Fibroblasts in each tissue microenvironment are adapted to cater for different cell types, but these are not badges of solid identity. They behave more like a verb rather than a noun.

Fibroblast heterogeneity does not only exist at the intertissue level, but also within tissues. For instance, secondary lymphoid tissues (Glossary) are enriched for lymphocyte-interacting fibroblasts such as fibroblastic reticular cells (FRCs) and follicular dendritic cells (FDCs). These fibroblasts generate chemokine gradients to recruit and compartmentalize lymphocytes into the T cell and B cell zones, respectively [25], but FRCs also help maintain the integrity of high endothelial venules [23]. In lymph nodes, barrier-forming functions are also required and provided by mesenchymal reticular cells (MRCs). MRCs support the lymphatic endothelial layer and macrophage niche of the subcapsular sinus, but they can also attract dendritic cells (DCs) to boost immune responses [23].

In peripheral tissues (Glossary) such as the synovium, synovial lining layer fibroblasts support a macrophage-driven barrier niche, aid the clearance of debris from the synovial space, and promote tissue remodeling. However, when activated, they drive cartilage destruction and bone damage. By contrast, sublining layer fibroblasts display a lymphocyte-interacting phenotype, recruiting and supporting lymphocyte accumulation during disease [18]. Overall, fibroblasts are now recognized as being specialized into a spectrum of phenotypes that differ not just in their roles but also their spatial localization within and between tissues.

### Location, location, location

#### Lymphoid tissues

**Fig 3:**
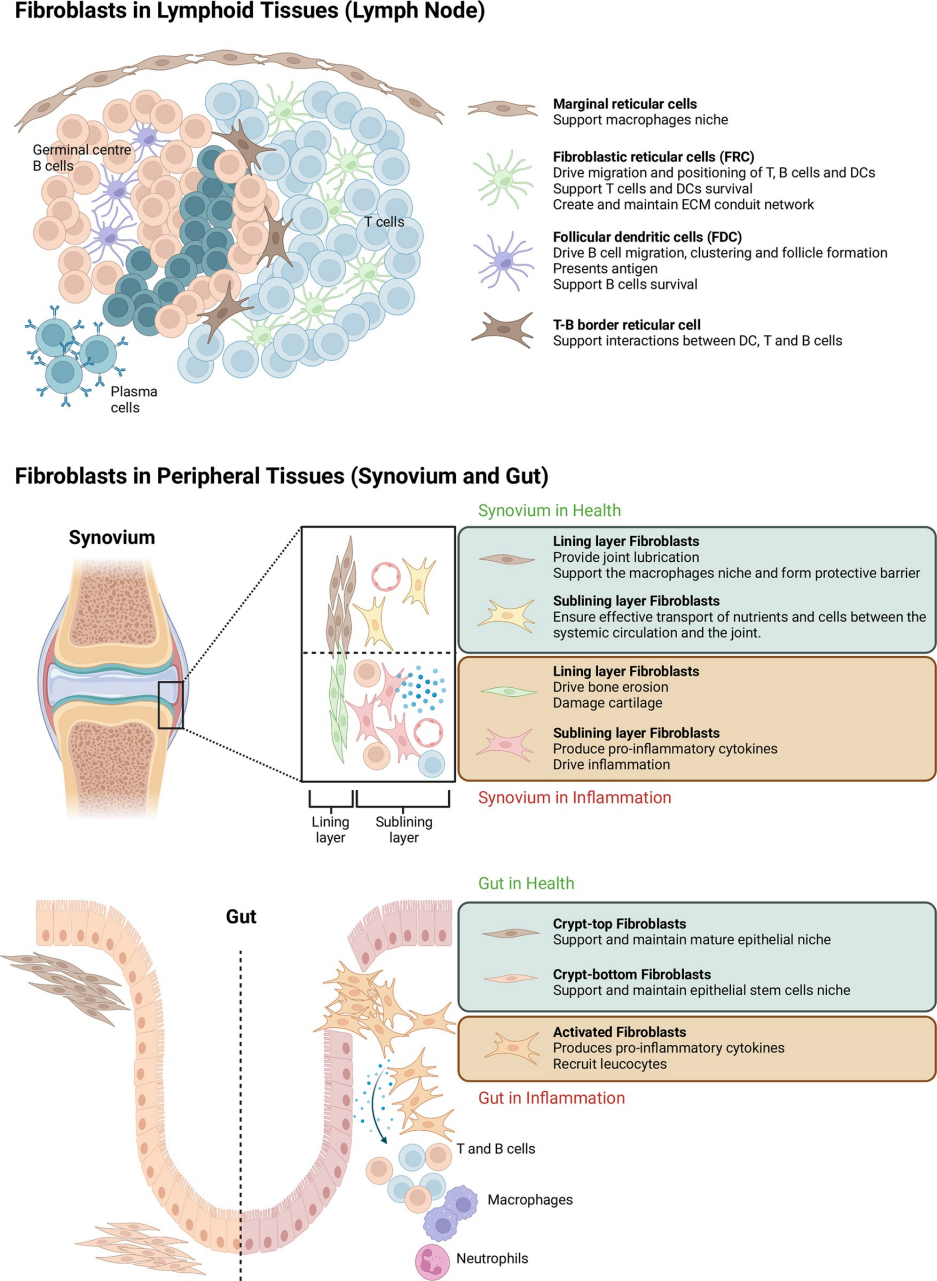
Fibroblasts in lymphoid and peripheral tissues. Fibroblasts have different roles in the lymphoid and peripheral tissues. They mainly define and maintain cellular niches within the tissue microenvironment to ensure smooth functioning of the tissues, but when they go rogue, they can drive tissue damage and orchestrate pathological inflammation. Figure is created with BioRender.com.

The specialization of fibroblasts is intrinsically linked to their positioning in space. Fibroblasts are sessile compared to leucocytes, and this places them in an ideal position to control the positioning and localization of cells within tissues. Secondary lymphoid tissues such as lymph nodes and spleen are prototypical examples of how fibroblasts control boundaries and help provide barriers within tissues. Within a naïve lymph node, B cells and T cells are highly segregated into well-defined areas, producing a baseline immunosuppressive environment by reducing communication between B cells and T cells that work together to trigger immunity. These spatial environments are produced by a series of distinct fibroblast populations that generate different chemokine gradients. For instance, FDCs localize B cells to the follicles through the expression of CXCL13, whereas T-zone reticular cells (TRCs) attract T cells and DCs to the T-zone by expressing CCL19 and CCL21. TRCs not only generate migratory cues, but they also produce growth factors to sustain immune cell niches and construct and maintain the ECM-rich conduit network so that small molecules can rapidly diffuse throughout the lymph node [25,26] (Fig 3).

In addition to lymph nodes, other specialized lymphoid tissues are found throughout the body, such as mucosal-associated lymphoid tissues in healthy gut and lung. In pathological conditions, tertiary lymphoid structures (TLSs) (Glossary) can form within peripheral tissues. Patterns of immune cell niches and chemokine networks observed in secondary lymphoid structures are broadly conserved in TLSs. During pathology, such as occurs in chronic inflammation, infection, and cancer, tissue-resident fibroblasts shift their phenotype to support leucocyte migration and TLS organization [27]. Given that TLSs are associated with worse outcomes in autoimmune diseases but with better survival in cancer, it is critical that the location and spectrum of fibroblast functions are tightly regulated. An open question that remains is: To what extent do the rules of intratissue patterning of immune cells by lymphoid tissue immune fibroblasts apply to peripheral tissues?

#### Peripheral tissues

As in lymphoid tissues, it is now clear that fibroblasts are imprinted with positional identity within peripheral tissues. For instance, gut fibroblasts located at the top of crypts promote epithelial differentiation, whereas fibroblasts at the bottom of crypts secrete factors that regulate maintenance, self-renewal, and differentiation of the stem cell niche (Fig 3) [21,28]. Similarly, in the synovium, synovial lining and sublining fibroblasts are two distinct populations, expressing different markers and holding different roles. For example, in rheumatoid arthritis (RA) (Glossary), lining fibroblasts drive cartilage destruction and bone damage, whereas the sublining fibroblasts are responsible for driving inflammation. New research findings suggest that lining and sublining fibroblasts might not be stable long-term clusters. Instead, they exist along a transcriptional gradient corresponding to their proximity to the vasculature and their anatomical location within the synovium [29]. Classically, lymphocytes are only found in the synovial sublining region in RA, where perivascular cuffing of lymphocytes is a well-known phenomenon observed at many sites of chronic inflammation. What confines lymphocytes to the sublining around perivascular microdomains and excludes them from the synovial lining remains unknown. As humans have evolved the ability to orchestrate precise localization and compartmentalization of immune cells in lymphoid organs, it is tempting to speculate that the peripheral organs also harness the same mechanisms to triage immune cells into different compartments.

Chronic inflammatory reactions have a predilection for certain anatomical sites. For example, RA occurs mainly in synovial joints and not the gut, whereas inflammatory bowel diseases (IBDs) (Glossary) occur in the gut and not the skin. Although these diseases are classified under the same umbrella of immune-mediated inflammatory diseases, we are only now starting to appreciate that fibroblasts in gut and joints can be very different and that this fibroblast heterogeneity may contribute to the site specificity. A good example is vitiligo, which is an autoimmune disease characterized by bilateral symmetric skin depigmentation. It has been a puzzle why vitiligo commonly presents on the face, neck, hands, and in skin creases. The answer has now been revealed by a recent piece of research that showed that anatomically distinct dermal fibroblasts have different abilities to recruit and activate cytotoxic T cells depending on their response towards cytokines (in this case, interferon gamma (IFNγ)), which in turn determines the patterns of vitiligo lesions on the skin [30].

#### When fibroblasts behave

In peripheral tissues, fibroblasts help maintain homeostasis, but they can also contribute towards pathology in disease states. Traditionally, fibroblast function has been thought to be limited to providing structural and biochemical support to surrounding cells by producing and remodeling ECM. Fibroblasts were thought to be invariant across tissues, despite the fact that, for some time, it has been clear that fibroblasts in different tissues produce distinct types of ECM with optimized mechanical properties best suited for the respective tissue function, for instance, respiration in the lung and gut motility [31,32].

Fibroblasts also have a central role in environmental sensing. Like macrophages, they act as sentinel cells and possess specialized sensory functions that detect changes in environmental factors such as mechanical forces, oxygen and nutrient levels [19]. In response to bacterial infection, intestinal fibroblasts produce chemokines that recruit pro-inflammatory monocytes and neutrophils, which in turn activates an adaptive immune response to eradicate the bacteria [33]. On the other hand, specialized kidney fibroblasts in the peritubular interstitium help maintain healthy systemic oxygen level by sensing hypoxia and producing erythropoietin as needed, which in turn stimulates red blood cell production in the bone marrow [34]. Fibroblasts also have a role in maintaining the correct cell numbers and ratio in each tissue compartment. As tissue density changes, fibroblasts sense the difference in mechanical force through actin-dependent mechanisms and then direct macrophage proliferation by regulating growth factor expression. In real time, cell loss due to injury or tissue damage results in increased space availability, and this is detected by the fibroblasts, which then increase macrophage numbers by increasing growth factor production [19,20]. While macrophages clear up the cell debris [35], fibroblasts work together with neutrophils to repair and regenerate the ECM, facilitating wound closure [36]. Given fibroblasts have important roles in the healthy behavior of tissues, it follows that their dysfunction should contribute to disease.

#### When fibroblasts go rogue

In disease, fibroblasts often acquire pathological features that facilitate disease progression, persistence, and recurrence at specific anatomical sites. One commonly pathological form of fibroblasts comes in the form of myofibroblasts (Glossary), a differentiated, contractile form of fibroblast that is found in tissue repair and fibrosis [37]. During wound healing, the myofibroblast phenotype is transient [38], but it becomes persistent in many fibrotic diseases, including idiopathic pulmonary fibrosis and Dupuytren’s disease [39,40]. The contractile and ECM-producing phenotype of myofibroblasts, as well as their pathogenic persistence, results in increased tissue stiffness in fibrotic tissues.

In addition to fibrosis, fibroblasts also contribute to chronic inflammation. Studies across multiple inflammatory diseases show that disease-related fibroblasts share common properties and communicate with both immune and vascular compartments, which then contribute to persistent inflammation [41]. In RA, synovial fibroblasts undergo metabolic reprogramming and mediate tissue priming by enhancing inflammasome activity, which means that sensitized synovial tissues are more prone to reinflammation, a concept that has been termed “stromal memory” [42]. More recently, in the case of Coronavirus Disease 2019 (COVID-19), a single-cell RNA lung atlas revealed higher fractions of pathological fibroblasts in the lungs of patients with COVID-19 as compared with healthy controls, with COVID-19 lung fibroblasts expressing collagen-producing and fibrosis-related signatures [43]. This insight would not have been possible without the latest advances in single-cell sequencing technology.

In the cancer field, researchers are starting to appreciate the importance of cancer-associated fibroblasts (CAFs) (Glossary), which are generally described as the fibroblastic population that accompanies solid tumors. They are complex, heterogeneous, and surprisingly plastic in their functional states. There are many potential sources of CAFs, including tissue-resident fibroblasts [44], bone marrow–derived fibroblasts [45], adipocytes [46], endothelial cells [47], and pericytes [48]. Not only do they arise from different sources, they also exist in different functional states and are described as inflammatory CAFs (iCAFs) [49], myfibroblastic CAFs (myCAFs) [49], antigen-presenting CAFs (apCAFs) [50], and vascular CAFs (vCAFs) [48]. MyCAFs tend to be found adjacent to the tumor and show a strong bias towards an ECM remodeling phenotype, whereas iCAFs tends to be more distal from tumor edge and express pro-inflammatory cytokines and chemokines that can activate and traffic immune cells [49]. On the other hand, apCAFs have the capacity to present antigens to T cells and promote immunity [50,51], and vCAFs are involved in vascular development and regulating angiogenesis [48]. Being the main cellular component of the tumor microenvironment for most solid tumors, CAFs have an important role in tumor initiation, progression, metastasis, local tissue metabolic reprogramming, and therapeutic resistance [11,12]. One of the open questions remains: Can we skew CAFs from an immunosuppressive to an inflammatory and immunomodulatory phenotype, which in turn could improve cancer immunotherapies?

#### Fibroblasts can swing between functional states

Cells can have both positional identity within tissues and a functional state (for instance, inflamed, replicating, or quiescent). As fibroblasts were thought to be mainly structural and inert, their functional states have until now not been well studied.

Fibroblasts in steady-state lymph nodes are fundamentally tolerance-promoting cells through a variety of mechanisms. In addition to segregating lymphocytes into distinct T cell and B cell niches to prevent unintended cell–cell interactions, fibroblasts also promote tolerance by presenting peripheral self-antigens. Lymph node fibroblasts also aid in the induction of regulatory T cells, both directly through TGF-β and indirectly by skewing DCs towards a tolerogenic phenotype [52,53]. This tolerogenic phenotype of lymph nodes varies across tissues, with mesenteric lymph nodes having a higher propensity towards tolerance promotion than skin-draining lymph nodes, most likely due to constant exposure to a high volume of gut microbiota.

Critically, lymph node fibroblasts have the capacity to transform the lymph node from a quiescent environment into one that supports highly efficient conditions for generating adaptive immune responses. Upon infection or immunization, lymph nodes can expand rapidly to up to 10 times their initial volume while maintaining their structural integrity [54]. The FRCs stretch and relax, which loosens the collagen-based reticular network, and this enhances the motility and interactions between immune cells [55]. Interactions between B cells and T cells at the border between the follicles and the T-cell zone are critical for the development of adaptive immunity. Cells are guided to this zone by chemokine gradients produced by fibroblasts that are laid down in the steady state [56]. Infection skews lymph node fibroblasts from tolerogenic towards an inflammatory phenotype with increases in antigen presentation, and chemokine and cytokine production [13].

Cytokine networks that drive pathogenesis in diseases such as RA and IBD are now well established [57,58]. However, the cellular origins of these cytokine culprits remain unclear. We need a better understanding of the cells producing the pro-inflammatory cytokines, such as tumor necrosis factor (TNF) and interleukin 6 (IL-6), so that we can target inflammatory disease at their cellular root. There is a consensus in the stromal field that fibroblasts in their activated states express fibroblast activation protein (FAP) and PDPN [59,60]. What regulates the switch between these distinct functional states is less clear, but pathogenic amplificatory loops involving fibroblast-driven regulatory networks, such as IL-6–JAK–STAT3, have been strongly implicated [17,41,57]. Could it be due to cytokines and growth factors produced by other tissue-resident cells such as macrophages, endothelial and epithelial cells in the microenvironment? As cellular metabolism is the cornerstone of all biological activities, could fibroblasts change their phenotype or functional state by undergoing metabolic reprogramming? With the latest tools and algorithms, such as COMPASS, we can not only sequence genes at a single-cell level, but are also now able to characterize their metabolism at the resolution of single cells [61], which should help in answering some of the questions posed above.

## Conclusions

Here, we presented fibroblasts as cells of many talents instead of featureless cells that are intimately involved in tissue homeostasis. Understanding the biology of fibroblasts builds our understanding of the role of the tissue microenvironment in disease and expands our knowledge from a purely reductionist view of the roles of individual cells, genes, and proteins to an integrated organ-centered view of pathology. Fibroblasts are vital within the tissue microenvironment because they define, maintain, and remodel their niches so that tissues can function in a homeostatic rather than maladaptive manner. Fibroblasts are also the puppeteers of our immune system and can orchestrate an immune response when necessary. Nevertheless, we still lack a deeper understanding of the interorgan heterogeneity of fibroblasts and how this relates to shared and divergent features of diseases. For example, we need to sample more cells from healthy tissues and compare them to cells from model organisms including mice and primates to relate mechanistic studies on fibroblasts to a human context. The Human Cell Atlas will attempt to complete this enterprise to deliver tangible dividends to medicine. The degree to which the rules of fibroblast–immune cell interactions in lymphoid tissues are conserved in peripheral tissues is also not fully known. We understand that fibroblasts in the periphery can display an immune-interacting phenotype (for instance, FAP^+^ fibroblasts in the gut), but to what degree their cellular crosstalk is the same as in lymphoid tissues is unknown.

As fibroblasts are increasingly recognized as being important in health and disease, this opens the possibility of targeting fibroblasts therapeutically in cancer, infection, and inflammatory diseases. Critically, our understanding of the interorgan and intraorgan heterogeneity of fibroblasts opens the possibility that we will be able to therapeutically target specific diseased tissues and cell states. Targeted depletion of fibroblasts has been achieved in the context of cancer by targeting FAP-expressing cells, but this led to adverse effects due to FAP expression in healthy tissues, especially bone marrow [62]. Given that these drugs were designed before the single-cell revolution, could our new understanding of fibroblast subsets help to design drugs with reduced off-target effects? Additionally, could the functional states of fibroblasts be targeted, and their biology skewed away from pathogenic to physiological states?

## Abbreviations

apCAF: antigen-presenting CAF
CAF: cancer-associated fibroblast
COVID-19: Coronavirus Disease 2019
DC: dendritic cell
ECM: extracellular matrix
FAP: fibroblast activation protein
FDC: follicular dendritic cell
FRC: fibroblastic reticular cell
IBD: inflammatory bowel disease
iCAF: inflammatory CAF
IFNγ: interferon gamma
IL-6: interleukin 6
ImmGen: The Immunological Genome Project
MHC: major histocompatibility complex
MRC: mesenchymal reticular cell
myCAF: myfibroblastic CAF
PDPN: podoplanin
RA: rheumatoid arthritis
scRNA-seq: single-cell RNA sequencing
TLS: tertiary lymphoid structure
TNF: tumor necrosis factor
TRC: T-zone reticular cell
vCAF: vascular CAF
